# Evaluation of the Prevalence and Risk Factors for Undernutrition in Hospitalized Dogs

**DOI:** 10.3389/fvets.2018.00205

**Published:** 2018-08-29

**Authors:** Jenifer Molina, Marta Hervera, Edgar Garcia Manzanilla, Carlos Torrente, Cecilia Villaverde

**Affiliations:** ^1^Fundació Hospital Clínic Veterinari, Universitat Autònoma de Barcelona, Bellaterra, Spain; ^2^Departament de Ciència Animal i Dels Aliments, Universitat Autònoma de Barcelona, Bellaterra, Spain; ^3^Expert Pet Nutrition, www.expertpetnutrition.com, Fermoy, Ireland; ^4^Animal and Grassland Research and Innovation Centre, TEAGASC Moorepark, Fermoy, Ireland; ^5^School of Veterinary Medicine, University College Dublin, Dublin, Ireland; ^6^Departament de Medicina i Cirurgia Animal, Universitat Autònoma de Barcelona, Bellaterra, Spain

**Keywords:** body condition score, body weight, dogs, frailty, hospitalization, undernutrition

## Abstract

Hospitalized dogs are predisposed to undernutrition, which can potentially affect outcome. This study evaluated the prevalence of undernutrition in hospitalized dogs (measured as loss of body condition score, BCS and body weight, BW) and studied the risk factors that affect nutritional status, and outcome (discharge or death). Data was collected prospectively during 9 months from 500 hospitalized dogs with a hospitalization length longer than 24 h in a veterinary teaching hospital in Spain. The BCS and BW changes were modeled using multiple linear regression and outcome was modeled using logistic regression. The risk factors studied were energy intake, hospitalization length, initial BW and BCS, age, sex, severity of clinical signs, department of admission, fasting or nutritional interventions, and the presence of anorexia, vomiting or diarrhea at admission. Most of the dogs (84.0%) consumed less than 25% of their energy requirements and only 3.4% of the dogs met these requirements. The majority of hospitalized dogs maintained their BCS (78.2%) and BW (77%) during hospitalization. Older patients (*P* = 0.040), higher initial BCS (*P* < 0.001), and vomiting at admission (*P* = 0.030) were associated with a decrease of BCS status during hospitalization. BCS was also decreased in patients with low energy intake, particularly in patients with hospitalization length longer than 3 days (*P* < 0.001). Both longer hospitalization length (*P* < 0.001) and vomiting at admission (*P* = 0.004) were also associated with a decrease in BW. Dogs that consumed their theoretical energy requirements [*P* < 0.001; Odds Ratio (OR) 0.95, 95% CI: 0.92 to 0.98], and had a higher initial BCS (*P* < 0.001; OR 0.39, 95% CI: 0.22 to 0.63) had a lower odds of dying. Anorexia at admission (*P* < 0.001; OR 5.67, 95% CI: 2.23 to 15.47) was associated with a higher risk of death. The results from this study support the finding that undernutrition is relatively common during hospitalization, with age, hospitalization length, decreased energy intake, and vomiting at admission as risk factors for undernutrition. Furthermore, an association was found between inadequate energy intake and death.

## Introduction

The importance of adequate nutrition in hospitalized dogs and cats is increasingly being recognized (1, 2). Hypermetabolism and reduced appetite, often found in ill animals, predispose these patients to undernutrition (3). Undernutrition can be defined in dogs and cats as BCS and/or BW below ideal. In the context of hospitalized dogs and cats undernutrition becomes a dynamic concept and is defined as involuntary loss of BCS and/or BW. Several studies have described undernutrition in hospitalized human patients with estimated prevalence from 25 to 50% or higher (4–7). There are very few studies assessing the prevalence of undernutrition in hospitalized dogs and cats. In one retrospective study (1) and two prospective studies (2, 8) the estimated prevalence of undernutrition in veterinary ranged between 25 and 65%.

There are also little data regarding which are the risk factors for undernutrition in hospitalized dogs and cats. Clinical signs such as vomiting, regurgitation, or abdominal pain have been proposed (9, 10). Animals presenting these risk factors had in general a worse outcome than those not affected (1, 7).

Nutritional support in hospitalized dogs and cats is key to providing the required energy and nutrients; avoiding metabolic disorders and protein catabolism; and maintaining normal organ functions (3). The lack of knowledge and well defined hospital protocols have been described as the main barriers to adequate nutrition in hospitalized human patients (11) and the same situation could be found in veterinary medicine (9). To identify undernutrition, a daily nutritional evaluation of hospitalized patients should be completed including BW, BCS, muscle condition score (MCS), dietary information, and environmental assessment (12).

Retrospective (7, 13) and prospective (14, 15) studies in human patients have reported that nutritional support is effective in improving the hospitalized patient status and reducing the hospitalization length (HL). There is a lack of research supporting the importance of nutritional support in dogs, although enteral feeding has been shown to be positive in dogs with acute gastrointestinal disease (16, 17). Early nutritional support has been associated with decreased HL in dogs with sepsis (18), and one study found an association between low energy intake (EI) during hospitalization and death (2). Another issue that has not been well defined for dogs is the best time to implement nutritional support (9).

The objectives of this prospective study were to assess the prevalence of undernutrition defined by loss of BCS and BW, to identify risk factors associated with undernutrition during hospitalization time, and to study the association of the nutritional status with outcome in hospitalized dogs.

## Materials and methods

A prospective cohort study was carried out in the hospitalization ward of a veterinary teaching hospital in Spain (Fundació Hospital Clínic Veterinari, Universitat Autònoma de Barcelona). Data from dogs was collected for 9 months (from April 2013 to January 2014) (Supplementary Material). All dogs with a HL longer than 24 h were included. The dogs were housed in the canine hospitalization ward, in individual kennels. An animal care and use protocol was approved by the Animal Protocol Review Committee of the Universitat Autònoma de Barcelona and owner consent was obtained to enroll the animals. Data collected from each animal included signalment, clinical signs at admission (anorexia, vomiting, and diarrhea), reason for hospitalization, diagnostics, fasting order at admission, nutritional evaluation at admission and for each day of the hospitalization period [BCS, MCS, and BW(kg)], HL (days), daily food type and intake (grams), type of nutritional intervention, and outcome (discharge or death). Clinicians from the corresponding department obtained histories and performed physical examination of the patients prior to hospitalization. The presence of weight loss, loss of appetite, vomiting, and diarrhea during the month before hospitalization was also identified by the clinicians during the history taking. Once the patient was hospitalized, the Nutrition Service personnel performed most of the nutritional evaluation. The same investigator (JM) performed all BCS and MCS measures.

Vomiting, anorexia, diarrhea and BW loss before admission reported by the owner were classified as “yes/no.” The reason for hospitalization and diagnostics was recorded as the clinical department in charge of the patient (surgery, internal medicine, neurology, ophthalmology, traumatology, emergency, and critical care). Severity disease was assessed using physical status score (PSS) as described by Brunetto et al. (1). The PSS is a 5-point scale where (1) indicates a normal animal with no organic disease, (2) mild systemic disease, (3) severe systemic disease limiting activity but not incapacitated, (4) incapacitating systemic disease that is a constant threat to life, and (5) a moribund animal not expected to live 24 h with or without any type of intervention. Fasting orders at admission (NPO) by the clinician were also classified as “yes/no.” Finally, the patient's outcome was classified as discharge or death.

The BCS was assessed using a 9 point scale where 1 is emaciated, 2 is very thin, 3 is thin, 4 is underweight, 5 is ideal, 6 is overweight, 7 is heavy, 8 is obese and 9 is morbid (19, 20). The MCS was assessed using a 4-point scale where 3 is normal muscle mass, 2 is mild muscle wasting, 1 is moderate muscle wasting, and 0 is severe muscle wasting (12).

Nutritional intervention (yes/no) was defined as any purposefully planned action decided by the Nutrition Service intended to meet the energy and nutrient requirements of a hospitalized animal. These interventions included feeding plans for voluntary oral feeding and enteral and parenteral nutrition support.

The average theoretical energy requirements were calculated for each dog using the resting energy requirements (RER) formula: RER = 70 × BW^0.75^ (21). The dog's daily EI was expressed as the average percentage of the RER consumed by the patient (calculated by dividing total energy intake by RER and multiplying this by 100). All dogs were offered initially a complete and balanced commercial diet decided by the clinician in charge of the case or by the Nutrition Service (if consulted). If the patient did not eat this option, the hospitalization ward personnel offered other commercial options with different texture and flavor. If none were accepted, they offered unbalanced homemade food (white rice and chicken breast cooked). If the attending veterinarian asked the Nutrition Service for a nutritional plan, the Nutrition Service calculated the specific requirements, food type, and food amounts for the dog daily. If the Nutrition Service was not consulted the ward personnel offered food without estimating requirements and recorded food type and amount. For patients that were anorexic or eating less than their RER after 3 days of hospitalization, the Nutrition Service suggested placing a feeding tube or initiating parenteral nutrition, although the final decision was made by the attending clinician.

## Statistical analysis

Data were analyzed using R 3.1.0 (R core team, RRID: SCR_001905). The dog was considered the experimental unit for all analyses. Descriptive statistics were performed for continuous and categorical variables. BCS change (BCS at admission—BCS at discharge or death), relative BW change [(BW at admission—BW at discharge or death)/BW at admission] during hospitalization, and outcome were considered as dependent variables. The main independent variables to be studied were EI and HL and the models also included iBW, iBCS, age, sex, PSS, department to which the dog was admitted, the recommendation of fasting or nutritional intervention and the presence of anorexia, vomiting or diarrhea at admission. Initially, a regression model (linear for BCS and BW change and logistic for outcome) was used where each predictor variable was included as a single fixed factor. Variables that had *P* < 0.25 in this univariable analysis were selected to build the multivariable model. Before entering the variables into a multivariable model, bivariate Pearson's and Spearman's correlations and Chi-square analysis were performed among independent variables in order to assess collinearity. Collinearity was further studied if the bivariate analysis showed a significant association and *r* > 0.70. In case of collinearity, the variable with the highest association with the dependent variable was entered in the model. If both variables presented similar associations with the dependent variable the selection was based on clinical criteria. Collinearity of the complete model was tested using the variance inflation factor (VIF). Linearity of the responses to each independent variable was checked graphically. The BCS change and BW change models were built using multiple linear regression with manual stepwise selection procedure. The outcome model was built using logistic regression with manual stepwise selection procedure and results are presented as Odds ratio (OR) and the 95% confidence interval (CI). All two-way interactions between significant variables in the multivariable models were tested. Model fit was assessed with the Akaike information criterion (AIC) and adjusted *r*^2^. Alpha level for determination of significance was 0.05.

## Results

Data were collected from 500 dogs and outcome was available for all 489 dogs; BCS change was recorded for a total of 445 dogs; and dogs were weighed (BW) by the hospitalization ward practitioners and nurses at discharge for 358 animals.

A detailed description of dependent and independent variables is presented in Tables 1, 2. The median age of the dogs was 6 years old (range: 2 months – 17 years) and the gender distribution was: 149 (30.5%) entire females, 104 (21.3%) spayed females, 193 (39.5%) entire males, and 43 (8.8%) neutered males. The mean initial BW (iBW) and initial BCS (iBCS) ± the standard deviation were 15.7 ± 12.5 kg and 5 ± 0.9 respectively.

**Table 1:** Descriptive statistics for continuous dependent and independent variables for the hospitalized dogs.

**Variable**	***n***	**Mean**	**SD^a^**	**Median**	**Minimum**	**Maximum**
**DEPENDENT VARIABLES**						
Body weight change	358	−0.2	0.5	−0.1	−2.2	2.2
Body condition score change	445	−0.1	0.4	0	−2	2
**INDEPENDENT VARIABLES**						
Age, years	481	6	4.3	6	0.16	17
Initial body weight, kg	489	15.7	12.5	12	0.4	75
Initial body condition score	470	5	0.9	5	1	8
Initial muscle condition score	470	3	0.6	3	0	3
Energy intake, %RER	487	34.5	36.7	23.9	0	210.8
Hospitalization length, days	489	4.1	2.8	3	1	20

a *SD, Standard deviation*.

**Table 2:** Descriptive statistics for categorical dependent and independent variables.

**Variable**	***n***	**Category: Frequency (percentage)**
**DEPENDENT**			
Outcome	489	Discharge: 453 (92.6%) Death: 36 (7.4%)
**INDEPENDENT**			
Sex	488	Entire Female: 149 (30.5%) Entire Male: 192 (39.4%) Spayed Female: 104 (21.3%) Neutered Male: 43 (8.8%)
Physical status score	470	1: 67 (14.3%) 2: 86 (18.3%) 3: 180 (38.3%) 4: 130 (27.6%) 5: 7 (1.5%)
Department in charge of the patient	482	Surgery: 22 (4.6%) Internal Medicine: 176 (36.5%) Neurology: 144 (29.9%) Ophthalmology: 46 (9.5%) Traumatology: 21 (4.4%) Emergency and Critical Care: 73 (15.1%)
Fasting ordered by clinician	488	Yes: 152 (31.1%) No: 336 (68.9%)	
Nutritional intervention	489	Yes: 152 (31.1%) No: 337 (68.9%)	
Anorexia at admission	473	Yes: 153 (32.3%) No: 320 (67.7%)	
Vomiting at admission	482	Yes: 79 (16.4%) No: 403 (83.6%)	
Diarrhea at admission	482	Yes: 40 (8.3%) No: 442 (91.7%)	

Regarding the nutritional status of the dogs, 77% of the hospitalized dogs maintained their BW during hospitalization, 16% lost BW and only 7% increased it. Gain or loss of BW was defined as a change of at least 5% from the patient's iBW. Similarly, most dogs (78.2%) maintained their BCS during hospitalization, while 18.4% lost, and 3.4% gained BCS. The median EI of hospitalized dogs was 23.9% of their RER ranging from 0 to 211%. Most of the dogs (84.0%) consumed less than 25% of their RER, whereas 7.1% consumed between 25 and 50%, and 5.5% consumed between 50 and 100%. Only 3.4% of the dogs consumed their RER or more. Therefore, most hospitalized dogs did not meet their energy requirements. Hospitalization ranged from 1 to 20 days with a median value of 3 days and 92.6% of the animals were discharged alive. The details for the distribution of patients within PSS categories and clinical departments and the proportion of animals with anorexia, vomiting, diarrhea, fasting and nutritional intervention are presented in Table 2.

Table 3 summarizes the associations between each risk factor and the dependent variables. Sex and diarrhea did not show association with any of the dependent variables. EI, HL, and PSS showed associations with the three dependent variables. The rest of covariates showed associations with 1or 2 of the dependent variables.

**Table 3:** Effects of the studied categorical risk factors for each of the dependent variables using univariable analysis.

**Variable**	**Change of ^a^BCS Mean ±SE^c^**	**Change of ^b^BW Mean ±SE^c^**	**Outcome death/Discharge, (%)^d^**
**SEX**			
Entire Female	−0.10 ± 0.02	−0.16 ± 0.05	13/136(8.7%)
Entire Male	−0.12 ± 0.03	−0.14 ± 0.05	11/181(5.7%)
Spayed Female	−0.06 ± 0.03	−0.08 ± 0.05	6/98(5.8%)
Neutered Male	−0.11 ± 0.04	−0.23 ± 0.12	6/37(14.0%)
*p*-value	*P* = 0.603	*P* = 0.886	*P* = 0.271
**PHYSICAL STATUS SCORE**			
1	−0.02 ± 0.02	0.01 ± 0.07	2/65(3.0%)
2	0.01 ± 0.04	−0.10 ± 0.08	4/82(4.7%)
3	−0.14 ± 0.04	−0.13 ± 0.04	11/169(6.1%)
4	−0.14 ± 0.04	−0.24 ± 0.05	13/117(10.0%)
5	−0.17 ± 0.11	−0.13 ± 0.08	3/4(42.8%)
*p-*value	*P* = 0.003	*P =* 0.054	*P* < 0.001
**DEPARTMENT IN CHARGE OF PATIENT**			
Surgery	−0.11 ± 0.05	0.03 ± 0.23	2/20(9.1%)
Internal Medicine	−0.15 ± 0.03	−0.09 ± 0.05	19/157(10.8%)
Neurology	−0.12 ± 0.03	−0.22 ± 0.04	7/137(4.9%)
Ophthalmology	0.03 ± 0.06	−0.05 ± 0.06	1/45(2.2%)
Traumatology	0.02 ± 0.06	−0.14 ± 0.06	0/21(0.0%)
Emergency and Critical Care	−0.08 ± 0.03	−0.21 ± 0.08	6/67(8.2%)
*p*-value	*P =* 0.032	*P* < 0.001	*P =* 0.208
**FASTING ORDERED BY CLINICIAN**			
Yes	−0.12 ± 0.02	−0.22 ± 0.05	30/306(8.9%)
No	−0.07 ± 0.03	−0.16 ± 0.08	6/146(3.9%)
*p*-value	*P =* 0.140	*P =* 0.383	*P =* 0.002
**NUTRITIONAL INTERVENTION**			
Yes	−0.07 ± 0.02	−0.16 ± 0.03	10/142(6.6%)
No	−0.17 ± 0.03	−0.09 ± 0.05	26/311(7.7%)
*p*-value	*P =* 0.009	*P =* 0.739	*P =* 0.795
**ANOREXIA AT ADMISSION**			
Yes	−0.08 ± 0.02	−0.13 ± 0.03	25/128(16.3%)
No	−0.14 ± 0.04	−0.12 ± 0.06	8/312(2.5%)
*p*-value	*P =* 0.134	*P =* 0.926	*P* < 0.001
**VOMITING AT ADMISSION**			
Yes	−0.21 ± 0.06	−0.15 ± 0.03	5/74(6.3%)
No	−0.08 ± 0.02	−0.14 ± 0.07	31/372(7.7%)
*p*-value	*P =* 0.004	*P =* 0.082	*P =* 0.407
**DIARRHEA AT ADMISSION**			
Yes	−0.14 ± 0.09	−0.15 ± 0.03	32/36(47.0%)
No	−0.10 ± 0.02	−0.06 ± 0.09	4/410(9.7%)
*p*-value	*P =* 0.454	*P =* 0.498	*P =* 0.567

a BCS, body condition score.

b BW, body weight.

c SE, Standard error.

d *Indicate the percentage of deaths for every category of each independent variable*.

**Figure 1:**
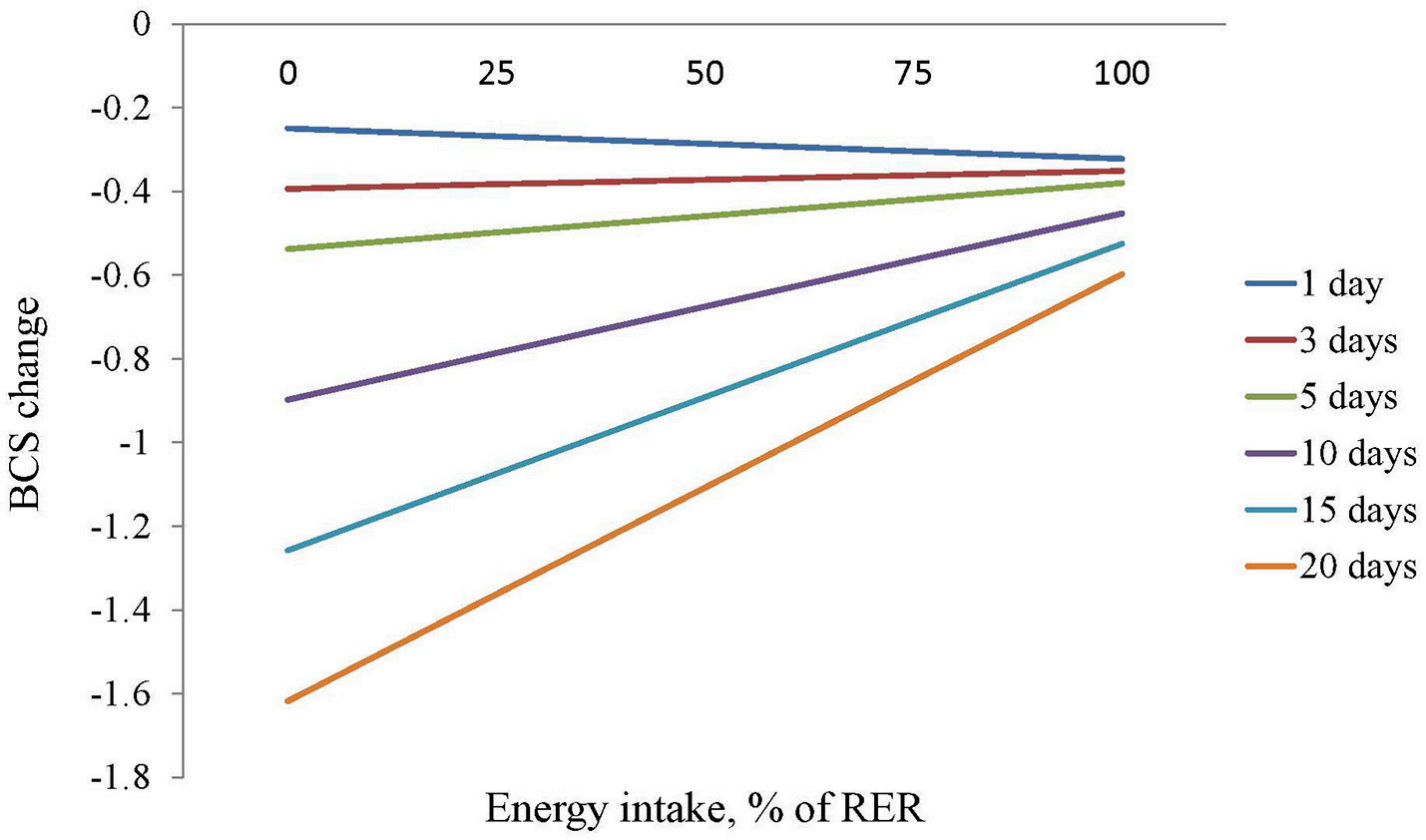
Estimation of the effect of the energy intake, measured as percentage of resting energy requirements, on BCS of dogs with different hospitalization lengths.

The multivariable analysis (Table 4) showed that older patients (*P* = 0.041), higher iBCS (*P* < 0.001) and vomiting at admission (*P* = 0.019) were associated to more severe BCS loss during hospitalization. There was also an interaction between HL and EI (*P* < 0.001) showing that a higher EI was associated with less BCS loss but only for HL longer than 3 days (Figure 1).

**Table 4:** Risk factors of the multivariable analysis for each dependent variable, body condition score change (ΔBCS), body weight change (ΔBW) and outcome.

	**Estimate**	**^a^SE**	***p*-value**
Δ**BCS (*****n*** = **445)**			
Intercept	0.4970	0.0936	<0.001
Hospitalization length (days)	−0.0820	0.0092	<0.001
Energy Intake (%RER)	−0.0017	0.0006	0.007
Age	−0.0074	0.0036	0.041
Initial BCS	−0.0562	0.0165	<0.001
Vomiting at admission	−0.0973	0.0411	0.019
Hospitalization length (days) × Energy Intake (%RER)	0.0007	0.0001	<0.001
Δ**BW (*****n*** = **358)**			
Intercept	−0.0229	0.0075	0.003
Hospitalization length (days)	−0.0035	0.0009	<0.001
Energy Intake (%RER)	0.0002	0.0007	0.008
Initial BW (Kg)	0.0008	0.0003	0.002
Vomiting at admission	0.0372	0.0128	0.004
Initial BW (Kg) × vomiting at admission	−0.0013	0.0059	0.033
**Outcome (*****n*** = **489)**	^b^ OR	95% CI ^c^	
Initial BCS	0.39	0.22, 0.63	<0.001
Energy Intake (%RER)	0.95	0.92, 0.98	<0.001
Anorexia at admission	5.67	2.23, 15.47	<0.001
Hospitalization length (days)	1.19	0.96, 1.45	0.092

a SE, Standard error.

b OR, Odds ratio; for continuous variables the OR indicate variation per unit.

c *CI, Confidence interval*.

Vomiting at admission, iBW and HL affected also BW change. Longer HL was associated with a greater loss of BW during hospitalization (*P* < 0.001). An interaction between iBW and vomiting (*P* = 0.033) was described, showing that vomiting was a risk factor for greater BW loss but mainly in heavier dogs.

Finally, the variables associated with outcome were iBCS, EI, anorexia at admission, and HL (Table 4). A higher iBCS and EI were related to lower odds of dying. In particular, the odds of dying were 61% lower for the heavier dog when 2 dogs differed in 1kg of iBCS (*P* < 0.001; OR 0.39, 95% CI: 0.22 to 0.63) and were 5% lower when 2 dogs differed in 1% RER of EI (*P* < 0.001; OR 0.95, 95% CI: 0.92 to 0.98). On the other hand anorexia at admission and longer HL were related to higher odds of dying. Dogs reporting anorexia at admission were at 5.67 greater odds of dying than those not presenting it (*P* < 0.001; OR 5.67, 95% CI: 2.23 to 15.47) and a dog was at 1.19 greater odds of dying than a dog with a HL 1 day shorter (*P* = 0.092; OR 1.19, 95% CI: 0.96 to 1.45).

## Discussion

There is a need to increase the body of knowledge in nutrition of hospitalized dogs and develop adequate protocols (9). The present study builds on the results of the only 3 studies available in this area (1, 2, 8) adding new risk factors of high interest, i.e. the presence of anorexia, vomiting and diarrhea at admission, and using a larger sample size. To the authors' knowledge, this is the largest prospective study assessing the prevalence of undernutrition and its risk factors in hospitalized dogs.

The present study assessed undernutrition in two ways: change of BCS and relative change of BW. We used both measures because BW can be rapidly affected by other aspects besides nutrition (such as fluid changes) and BCS is a much more consistent measure over short periods of time. It is estimated that there needs to be a 10-15% change in BW to see a change in one BCS unit (19). If we consider loss of more than 5% of BW and loss of BCS as undernutrition, the prevalence of undernourished dogs during hospitalization in this study was close to 20%. Brunetto et al. (1) reported similar results; using BCS change, the prevalence of undernourished dogs was around 20%. However they reported 46 % prevalence using BW change, a higher proportion than in the present study, probably because they considered 2% as their cut-off value for a significant change in BW. If we apply such cut-off to the present study the percentage of undernourished dogs is almost 40%, however the authors consider that 2% may be too low of a cut-off because such variation may be the consequence of changes in hydration or meals.

Regarding the EI, 96% of the dogs did not meet their estimated energy requirement. This suggests that most of the hospitalized population were at risk of undernutrition and might have resulted in BW and BCS losses with longer HL. Previous studies found lower percentages; 65% for (1) and 73% for (2), respectively. In the case of Brunetto et al. the proportion of animals gaining weight was also higher than in our case (40 vs. 7%), which might be related to the higher number of animals that met their estimated energy requirements. The difference with (2) could be due to a difference in experimental units. We used the average EI of all the hospitalization period while Remillard et al. used the daily EI per dog. Dogs that had an average negative energy balance during the hospitalization period may actually meet their requirements some of the days.

The undernutrition risk factors identified in this study were consistent within the different undernutrition estimators. EI and HL were significant in all three models and vomiting at admission and iBCS were significant in 2 models as shown in Table 4.

As expected, EI was positively associated and HL negatively associated with BW and BCS change. There was an interaction between HL and EI, based on the results of the BCS model, showing that EI becomes critical in preventing loss of BCS in animals with HL longer than 3 days. Patients are able to maintain their body mass for a few days even facing anorexia, however nutritional support would be needed in patients with expected HL of 3 days or longer as it has been proposed as a general rational rule in veterinary practice (9, 22). We also found that EI was positively associated with better outcomes, a finding also reported by Brunetto et al. and Remillard et al. (1, 2) as well as in human medicine (6, 13, 14). We did not find an association between PSS and BW or BCS change or outcome, which suggests that the protective effect of EI is not associated with disease severity. However, the small number of animals that died or were euthanized, plus the lack of a standardized objective severity index (23) in our study limits the interpretation of these findings.

An association was found between iBCS and BCS change during hospitalization. Heavier dogs at admission lost more BCS than did thin dogs. We hypothesize that veterinarians may pay more attention to the nutritional support of thin animals compared to heavier dogs, because they appear to be at immediate nutritional risk. However, BW change, which is a more objective measure, was not related to iBCS. Despite this finding, iBCS was positively correlated with discharge as also reported (1). Rather than reflecting a protective effect of obesity, we believe this finding supports that underweight dogs are at higher risk of dying than dogs with adequate body stores. In some cases, this lower BCS may be related to the course of disease before admission and here a more accurate measure of disease severity may be needed.

Vomiting at admission was also associated with a deterioration of nutritional status measured as BCS and BW change. In the case of BW change, vomit had an interaction with iBW, showing differences in vomiting effects on BW depending on dog size. In the present study, iBW was included as a measure of the size of the animal to avoid loss of information by categorization. In a closer look to the relationship between iBW and BW change, dogs heavier than 35 kg at admission appeared to lose more weight during hospitalization, especially when vomiting was present. We hypothesize that the food amount offered by the hospitalization ward personnel was smaller in large dogs than in the small ones, especially if vomiting was their reason for hospitalization, or that a specific cause of admission was typical of this group. Unfortunately, our data cannot verify the hypothesis. Future research should study the nutritional requirements of hospitalized dogs of extreme body size for a more accurate nutritional support.

Age was identified as a risk factor for undernutrition only for BCS change. Older dogs had a higher BCS loss than the younger patients. This could be due partly to the potential effect of aging on digestive physiology (24), thus ultimately affecting nutrient digestibility (25). Nevertheless, several studies have failed to demonstrate differences in macronutrient digestibility between young and old healthy dogs (26, 27) and some have even reported an increased nutrient apparent fecal digestibility in older dogs (28). Higher severity of disease or comorbidities could be another explanation for the greater BCS loss in older dogs however; our data cannot confirm this hypothesis either. In this sense, older dogs may suffer of frailty making them more vulnerable to stressors or disease than younger dogs (29). Some of the risk factors included in this study such as anorexia or iBCS are related to frailty and this concept could easily be included in future research by using one of the numerous available tools.

Finally, anorexia at admission was associated with higher risk of death, which may be related to its high correlation to severity of disease. During the statistical analysis, anorexia had a better correlation to outcome than severity, which is why it was kept in the model. It seems clear that anorexia is a main risk factor for death, reinforcing the need of nutritional support in those patients during the hospitalization period.

The clinical department in charge of the patient was initially included in the study as a representation of the nature of the type illness. However this variable had multiple associations to other risk factors and was finally not included in the models. The specific protocols used in each clinical department can be very different between hospitals and these results may not be fully valid for other hospitals. These protocols are also a potential source of bias because some departments may be more likely to recommend nutritional support than others.

The main limitations of the present study were the low number of patients who died or were euthanized, the limited number of enteral and parenteral nutrition support cases, the lack of an objective measure of severity like the acute patient physiologic and laboratory evaluation (APPLE) and the fact that it is a single center study. The number of dogs that die or receive nutritional support may differ between hospitals due to differences in protocols and in the type of animals that are hospitalized. For example, Brunetto et al. (1) reported a similar mortality (7%) but Remillard et al. (2) reported a much higher mortality (16%) compared to the present study. These differences between hospitals may also result in differences in the prevalence of undernutrition and in the importance of each risk factor. Studies comparing these results between hospitals would be of great interest. Remillard et al. was a multicenter study however there was no comparison of the different hospitals involved. Furthermore, the low number of dogs with nutritional support made it impossible to study its effects. More specific studies focused on cases of enteral and parenteral nutrition support are needed to properly estimate their effects. Finally, in the present study we used a subjective measure of severity (PSS) to be able to compare our results to those obtained by Brunnetto et al. and Remillard et al. Future studies should include more objective measures of severity like the APPLE (30) in order to study the actual effect of physiological status on EI and outcome.

## Conclusions

The results from this study support the findings that undernutrition is relatively common in hospitalized patients, and that there is an association between inadequate EI and undernutrition and negative outcome. The study also reports that long hospitalization periods are a risk factor for undernutrition and poor outcome. Old age, larger size, vomiting at admission and higher iBCS were identified as risk factors for undernutrition. Future prospective studies with a higher number of patients are indicated in order to evaluate the association between assisted nutrition and outcome in critically ill animals.

## Author contributions

CV and MH designed the study. JM with the help of CV and CT carried out the study. EM analyzed and interpreted the data. JM drafted the manuscript. CV, MH, and EM read and helped with corrections in the manuscript. All authors read and approved the final manuscript.

### Conflict of interest statement

Authors CV and MH were affiliated to the Departament de Ciència Animal i dels Aliments, Universitat Autònoma de Barcelona, Bellaterra, Spain at the time of the experiment, and they are currently employed by the consulting company Expert Pet Nutrition. The remaining authors declare that the research was conducted in the absence of any commercial or financial relationships that could be construed as a potential conflict of interest.

## Abbreviations

BCS: body condition score
BW: body weight
EI: Energy intake
HL: hospitalization length
iBCS: initial body condition score
iBW: initial body weight
MCS: muscle condition score
PSS: physical status score
RER: Resting energy requirements
ΔBCS: body condition score change
ΔBW: body weight change.
