# Supplementary material for: Evaluation of the Prevalence and Risk Factors for Undernutrition in Hospitalized Dogs
Source: Front Vet Sci. 2018 Aug 29;5:205. doi: 10.3389/fvets.2018.00205 (PMC6123354; doi:10.3389/fvets.2018.00205)
Supplement: Supplementary file 1 [file Table_1.DOCX]

Supplementary Material

**Evaluation of the prevalence and risk factors for undernutrition in hospitalized dogs**

**Jenifer Molina, Marta Hervera, Edgar Garcia Manzanilla, Carlos Torrente Artero, Cecilia Villaverde***

*** Correspondence:** Corresponding Author: [**contact@expertpetnutrition.com**](mailto:contact@expertpetnutrition.com)

# Supplementary Data

Supplementary data file: “Raw data_Villaverde 2018_Suppl.Material”

Variables in the raw data:

- HL: Hospitalization length, measured in days.
- EI: Energy intake, expressed as the average percentage of the Resting Energy Requirements (RER) consumed by the patient.
- Sex: Classified by numbers:
  - 1: Female
  - 2: Male
  - 3: Spayed female
  - 4: Neutered male
- Outcome:
  - 0: Discharged
  - 1: Dead
- iBW: Initial Body weight, measured in Kg.
- ΔBW: Body weight change, measured in Kg.
- iBCS: Initial Body condition score, assessed using a 9 point scale where 1 is emaciated, 2 is very thin, 3 is thin, 4 is underweight, 5 is ideal, 6 is overweight, 7 is heavy, 8 is obese and 9 is morbid (Laflamme, 1997a; Laflamme, 1997b).
- ΔBCS: Body condition score change.
- iMCS: The MCS was assessed using a 4-point scale where 3 is normal muscle mass, 2 is mild muscle wasting, 1 is moderate muscle wasting, and 0 is severe muscle wasting (Freeman et al., 2011).
- ΔMCS: Muscle condition score change
- Nutritional intervention: defined as purposefully planned actions decided by the Nutrition Service intended to meet energy and nutrient requirements of a hospitalized animal.
  - 1: Yes
  - 2: No
- NPO: Fasting ordered by clinician?
  - 1: Yes
  - 2: No
- Anorexia at admission
  - 1: Yes
  - 2: No
- Vomiting at admission
  - 1: Yes
  - 2: No
- Diarrhea at admission
  - 1: Yes
  - 2: No
- PSS: Physical status score, measured in 5-point scale.
  - 1: Normal animal with no organic disease.
  - 2: Mild systemic disease.
  - 3: Severe systemic disease limiting activity but not incapacitated.
  - 4: Incapacitating systemic disease that is a constant threat to life.
  - 5: Moribund animal not expected to live 24 hours with or without any type of intervention.
